# Genetic Analysis Workshop 13: Simulated longitudinal data on families for a system of oligogenic traits

**DOI:** 10.1186/1471-2156-4-S1-S3

**Published:** 2003-12-31

**Authors:** E Warwick Daw, John Morrison, Xiaojun Zhou, Duncan C Thomas

**Affiliations:** 1 Department of Epidemiology, University of Texas M.D. Anderson Cancer Center, Houston, Texas; 2 University of Southern California, Los Angeles, California, USA

## Abstract

The Genetic Analysis Workshop 13 simulated data aimed to mimic the major features of the real Framingham Heart Study data that formed Problem 1, but under a known inheritance model and with 100 replicates, so as to allow evaluation of the statistical properties of various methods. The pedigrees used were the 330 real pedigree structures (comprising 4692 individuals) with some minor changes to protect confidentiality. Fifty trait genes and 399 microsatellite markers were simulated by gene dropping on 22 autosomal chromosomes. Assuming random ascertainment of families, a system of eight longitudinal quantitative traits (designed to be similar to those in the real data) was generated with a wide range of heritabilities, including some pleiotropic and interactive effects. Genes could affect either the baseline level or the rate of change of the phenotype. Hypertension diagnosis and treatment were simulated with treatment availability, compliance, and efficacy depending on calendar year. Nongenetic traits of smoking and alcohol were generated as covariates for other traits. Death was simulated as a hazard rate depending upon age, sex, smoking, cholesterol, and systolic blood pressure.

After the complete data were simulated, missing data indicators were generated based on logistic models fitted to the real data, involving the subject's history of previous missing values, together with that of their spouses, parents, siblings, and offspring, as well as marital status, only-child indicators, current value at certain simulated traits, and the data collection pattern on the cohort into which each subject was ascertained.

## Background

Our goal in simulating data for Genetic Analysis Workshop 13 (GAW13) was to provide a data set with the basic features of the real data [1], a set of families from the Framingham Heart Study (FHS) [2], but under a known "true" inheritance model. The Framingham study has a number of unique features, but those we focused on replicating in our simulated set were the longitudinal collection over many years of several related traits on a large set of pedigrees and the availability of a complete genome screen with microsatellite markers. There has been a rapidly growing statistical literature on the analysis of dependent data, including longitudinal data, but seldom have genetic analyses addressed simultaneously the complexities of dependencies both within individuals over time and between individuals within pedigrees. Longitudinal data pose additional challenges with potentially informative missingness. This simulated set allows studies of false-positive rates and power for methods that might be applicable to the real data. It was our intention to encourage comparisons between results from the real and simulated sets, in the hope that some groups would find both sets useful in developing new methods.

To facilitate the use of both real and simulated data together, the simulated data set contains variables with the same names and in the same format as the real data. As with the real data, the simulated data contains measures of height (*HT*), weight (*WT*), high density lipoprotein (*HDL*), total cholesterol (*CHOL*), triglycerides (*TG*), glucose (*GLUC*), systolic blood pressure (*SBP*), hypertension diagnosis and treatment (*T*), cigarettes smoked per day (*SMK*), and quantity of alcohol consumed per week (*DRINK*). These variables were simulated longitudinally on two cohorts drawn from 330 pedigrees containing 4692 individuals, with data collection on each cohort starting about 30 years apart. The first cohort was examined 21 times at 2-year intervals, while the second was examined 5 times with an 8-year interval between the first two exams and 4-year intervals between subsequent exams. A missing data pattern was simulated to mimic that seen in the real data. To avoid any potential confusion with the real data, the placement of some individuals within some pedigrees was changed and all the sexes were randomized.

**Figure 1:**
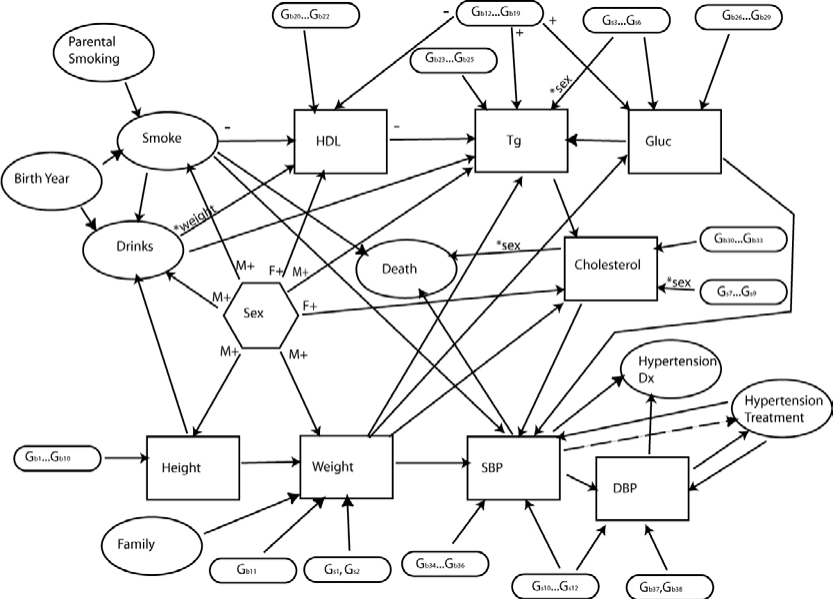
**Diagram of relationships between simulated traits and genes. **Arrows indicate causal relationships between traits. Most correlations are positive, but a "-" indicates a negative correlation. An "*" and trait name next to an arrow indicates that the relationship is mediated by the named trait.

Underlying the phenotype simulation, we simulated 449 genetic loci on 22 autosomal chromosomes via random gene drop. These included 399 microsatellite markers and 50 trait loci. We used a sex-specific map — another first for a GAW simulation — and the allele frequencies of the markers provided for the Framingham Heart Study data. The trait loci were randomly placed, but some chromosomes were excluded from having loci placed on them, so false-positive rates could be assessed. The 50 trait genes fed into a complex model (Figure 1), with some genes affecting the "baseline" trait value, and others affecting change in the trait over time. Some genes directly affect only one trait; others affect several. Some effects of these trait loci are large and easy to detect, some are smaller and more difficult to detect, and some are so small we expect them to be impossible to detect in a single replicate. We included genes of miniscule effect both to add a degree of realism to the simulation and in the hope that our expectation will be proven wrong.

Despite the complexity in this model, we are under no illusion that we met the impossible goal of exactly modelling the unknown biological mechanisms underlying these traits. Our primary concern was to provide a data set with a variety of different types of effects. These effects are designed to help us understand what types of genetic effects can and cannot be detected in a real data set like that collected in the Framingham study. In deciding what effects to include, we gave consideration to the correlations in the real data and the advice of biologists. We placed some limits on the types of models we considered: for reasons of limited human and computer time, we excluded any models that contained feedback loops, which almost certainly exist between some of these traits in reality: it is possible that increasing cholesterol levels contributes to weight gain, which in turn contributes to higher cholesterol levels, but this type of interaction was not included. Similarly, we only allowed the genetic effects to interact with each other and the environmental effects additively or multiplicatively: we felt it was more important to focus our time on the longitudinal aspects of the model. This simulated data set is designed to aid in the testing of methods, not to illustrate the workings of the human body.

Although our simulation is far less complex than reality, there is still much complexity in the model. We have interacting environmental variables: smoking is affected by parental smoking and birth year, while drinking is affected by smoking and birth year. Both the criteria for hypertension treatment and the treatment itself change with time. There are both direct sex effects and sex-moderated effects. The 50 loci we included represent nearly an order of magnitude more than have been included in previous GAW simulations. Of the eight simulated traits that were directly affected by trait loci, weight had the smallest number directly affecting it, with three, and glucose the most, with 16. Some trait loci had direct effect on two or three traits. When indirect effects are considered as well, the picture is even more complex: all 50 trait loci have an effect, direct or indirect, on hypertension diagnosis and DBP. We expect that this data set contains far more complexity than can be detected, even if all 100 simulated replicates of the data are analyzed simultaneously. In this respect, we believe that this simulated set does mirror reality.

## Methods

For this simulation, we broke the process into several parts. First, we constructed pedigrees based on those in the Framingham study, along with simulated birth years. This was followed by simulation of genotypes via random gene drop. These genotypes and the pedigrees were then fed into a program to generate the longitudinal data. Finally, a missing data program was run to determine which variable values were observed, including whether each individual was a member of Cohort 1, Cohort 2, or neither.

### Pedigree structures

The pedigrees and individual birth years were the same for all 100 replicates of the simulated data. We took the pedigrees provided by the Framingham Heart Study for GAW and randomized the sexes. In an effort to further obscure any connection between the real and simulated pedigrees, in a few of the larger pedigrees, we deleted an individual from one sibship and added an individual to another sibship within the same pedigree, thus maintaining the same sample size. The sample contained 4692 individuals in 330 families containing 7 to 84 people. Two of the families contained marriage loops. Because the ascertainment and missing data part of the simulation contained a random component, the number of individuals genotyped and phenotyped differed slightly between replicates: in the first replicate, which was the one GAW participants using only a single replicate were advised to use, 1720 individuals were genotyped and 2860 had some phenotype information available. We approximated the ages in the real data by assuming the first visit for the "original" cohort occurred in 1948 and the first visit for the "offspring" cohort occurred in 1972. We used these approximate ages to obtain distributions for age differences between mother and first child for sibships of different sizes, between set of sibs for sibships of different sizes, and for husband-wife age differences. We discarded some outliers from these distributions and then used them to randomly assign ages to the pedigrees by selecting a reference individual for each pedigree in a sibship without offspring, assigning that individual a birth year between 1937 and 1942 (uniform distribution), and spanning the pedigree by drawing randomly, with replacement, from the age difference distributions. If all individuals in a pedigree were born after 1952, or at least one individual was born after 1935 in a sibship without offspring, then random age assignment was repeated until obtaining one that met the requirements. Four pedigrees failed to have ages assigned after 500 iterations of the process, so we took one of the random age assignments for each of these four pedigrees and modified it so that it passed the rejection criteria. It was because of the complexity we encountered in this age assignment process and the limited time available that we decided to use the same ages for all 100 replicates.

### Marker and trait genotypes

The genotype simulation was done with the Genedrop program in the PANGAEA: Morgan package . The simulated markers are based on those available in the real data, which was genotyped using the Marshfield mapping panel 8A. We used the sex-specific Haldane map distances obtained by converting the maker positions found at the Marshfield web site  from Kosambi to Haldane centimorgans. The allele frequencies used in the simulation were those provided to us by the Framingham Heart Study group. We assumed Hardy-Weinberg and linkage equilibrium between all loci, both trait and marker. The 50 simulated trait genes were randomly placed on the odd-numbered chromosomes (Table 1), leaving the even-numbered chromosomes available for false-positive studies. Initially, we simulated the trait genes with 20 equally frequent alleles to give us flexibility in assigning those genes to different traits with different allele frequencies. These 20 alleles were reduced to two, three, or four alleles when we generated the trait values.

To generate the longitudinal data, we simulated data points for each individual in every year, from the time they were 20 until they reach 100. In all the trait models we used here, we restricted genes to be one of two types: "baseline" genes have a constant absolute effect on the trait value over time, while "slope" genes have a changing effect over time. In terms of percentage variance contribution to the trait, baseline genes will tend to have a decreasing effect with time, while slope genes will have an increasing effect. The numerical effects of each genotype at each locus are given in Table 2. Note that the variance given for each locus is the absolute variance for that locus rather than the proportional variance contribution: we include these numbers as a guide for judging the relative importance of each locus within a trait. Although the trait model itself is complex (Figure 1), it has a flow to it, and we cover the simulation of the trait values in that order.

The genotype simulation did not allow for any pedigree or genotyping errors and treated the map function as correctly specified. Likewise in what follows, no errors were simulated in the phenotype data, other than missing data.

### Phenotypes

#### Height

Height (*HT*) is a "simple oligogenic" trait, in that all effects are additive and there is no time dependence other than random noise. The two sexes *s *= (*m*, *f*) were given different means and variances: we used the values from the real data: μ_*HT *_(*m*) = 68.13 in, μ_*HT *_(*f*) = 62.57 in, σ_*HT *_(*m*) = 3.02 in, σ_*HT *_(*f*) = 2.70 in. Ten loci contribute to this trait, *G*_b1_,...,*G*_b10_, with contributions to the sex-specific variance of 40%, 20%, 10%, 5%, 2%, 2%, 2%, 1%, 1%, and 1%. In addition, a random environmental effect (constant over age) *r*_*HT *_contributes 14% of the sex-specific variance and an age-specific random effect at age *a *("measurement error"), ε_*HT*_(*a*), contributes 2% (both normally distributed). The formula used was:



where *g*_*bi *_is the effect of each individual's genotype at locus *G*_*bi *_(Table 2).

#### Weight

We chose to make weight (*WT*) strongly dependent on height via a BMI-like relationship, i.e., proportional to height in meters squared. Weight was simulated in pounds and height in inches, so some unit conversion was required. Since a substantial number of studies have reported localizations of genes influencing BMI in real data, we wanted to provide a simulated data set in which analysis of the simulated BMI could reasonably be expected to localize some of the simulated trait loci. All the loci that contribute to variation in height contribute indirectly to variation in weight through the height variable. In addition, one locus contributes to baseline weight, *G*_*b*11_, and two contribute to a logarithmic change in weight with age, *G*_*s*1 _and *G*_*s*2_.

*WT*(*a*) = (*HT*(*a*))^2 ^(*B*_*WT *_+ *S*_*WT *_log_10_(*a*)) + ε_*WT *_(*a*),

where *B*_*WT *_= α(*s*) + 10(*g*_*b*11 _+ *r*_*WT, fam *_+ *r*_*WT*,1_),

*S*_*WT *_= 2+ 2 (*g*_*s*1 _+ *g*_*s*2 _+ *r*_*WT*,2_).

In this equation, we converted height to meters before squaring it, and α(*s*) = 57 pounds/(meter)^2 ^for males and 53 for females, *r*_*WT*,*fam *_a random effect for family, and *r*_*WT*,1 _and *r*_*WT*,2 _time-constant normal deviates. The baseline gene contributes 40% of the variance in sex-specific baseline (*B*_*WT*_), family contributes 30% of sex-specific baseline variance, and *r*_*WT*1 _contributes 30% of sex-specific baseline variance. The slope genes contribute 50% and 40% of variance to the multiplier (*S*_*WT*_), and *r*_*WT*,2 _contributes 10%. ε_*WT *_(*a*) was a *N*(0, √3) random number.

#### Smoking and drinking

Both smoking and drinking were simulated as "environmental" variables (i.e., none of the simulated genes had a direct effect on either variable). We used patterns observed in the real data and from Johnson and Gerstein [3] as guides in simulating these variables. We found that the frequencies from Johnson and Gerstein did not produce the smoking and drinking rates observed in the real data, so we followed the trends in that paper while increasing the rates. We assumed that children of smokers were twice as likely to smoke as children of non-smokers and that smokers were 10% more likely to drink than non-smokers, to produce a sex by cohort probability table (Table 3). The probability of smoking depends on parental smoking. The probability of being a drinker depends on whether an individual is a smoker. We sampled from real-sample sex-specific distributions of drinks and cigarettes to get the simulated quantities. For drinkers, this sampled value was modified by height, so the quantity consumed has a genetic component of variation through height, but whether or not someone drinks does not. We chose to make the quantity consumed dependent on height, based on observing such a correlation in the real data. The correlations with BMI and weight were not as strong. Thus:

*DRINK *= *d*_*r *_((*HT*/μ_*HT *_(*s*)) + 1)/2,

where *d*_*r *_is the drink quantity (in grams per day), sampled from the sex-specific distribution, *HT *is height (without the measurement error term), and μ_*HT*_(*s*) is the sex-specific mean height. Quantities for both smoking (*SMK*) and drinking were fixed over time, but smokers have a probability (*a *- 20)/1000 of quitting each year, so *SMK*(*a*) denotes the current number of cigarettes smoked per day. We also calculated pack-years (*PY*), assuming all smokers started at age 18.

#### Lipids

High density lipoprotein (*HDL*), triglycerides (*TG*), and glucose (*GLUC*) have eight baseline genes in common, *G*_*b*12_,...,*G*_*b*19_, and *TG *and *GLUC *have four slope genes, *G*_*s*3_,...,*G*_*s*6_, in common as well. In addition, three baseline genes (*G*_*b*20_,...,*G*_*b*22_) only contribute directly to *HDL*, three (*G*_*b*23_,...,*G*_*b*25_) only contribute to *TG*, and four (*G*_*b*26_,...,*G*_*b*29_) only contribute to *GLUC*. *HDL *and *TG *have direct sex effects, and, in addition, the effects of *G*_*s*2_,...,*G*_*s*6 _on *TG *are mediated by sex via a menopause-like effect in women. All three were simulated to correspond to the measurements in units of mg/dl found in the real data. Weight has an effect on *TG *and *GLUC*, drinking on *HDL *and *TG*, and smoking on *HDL*. *HDL *and *GLUC *also have a direct effect on *TG*. All these effects were consistent with correlations seen in the real data. The equations used were:



where μ_*HDL*_(*s*) = 41 mg/dl for men and 53 mg/dl for women, σ_*HDL*_(*s*) = 11 mg/dl for men and 12 mg/dl for women,  is the baseline *DRINK *effect on *HDL*, where *WT(a) *is weight with no error,  is the baseline *SMK(a) *effect on *HDL*, and *r*_*HDL *_and ε_*HDL*_(*a*) are independent fixed (mean 0, variance 0.1) and age-varying normal deviates, respectively. The genetic effects on *HDL*, *g*_*Hbi*_, are given in Table 2. While *HDL *has no slope term, the presence of weight in the drinking term and people quitting smoking will cause some age dependence.

Both *GLUC *and *TG *have exponential slope terms, but *TG *also has a "menopause effect" in the slope term.



where  is the glucose baseline term, with *g*_*Gbi *_the effect of the genotype at locus G_bi _on *GLUC *(Table 2), *WT(a) *the weight without error, μ_*WT,s *_the sex-specific mean weight (176.03 pounds for men, 144.12 for women), and *r*_*GLUC*,1 _~ *N*(0, √0.1),



is the glucose slope term with *g*_*Gsi *_the effect of the genotype at locus *G*_*si *_on GLUC and *r*_*GLUC*,2 _~ *N*(0, √0.003). The glucose slope term can be positive or negative and is small in absolute value.

Triglycerides had baseline levels depending on *DRINK*, *HDL*, *GLUC*, and *WT*, together with 11 genes, and a slope that depended upon four genes, modified in females by a complicated function of age and age at menopause, which was also random:



where μ(*s*) = 75 mg/dl or 65 mg/dl and σ(*s*) = 25 mg/dl or 20 mg/dl for males or females, respectively, *g*_*Tbi *_is the effect of the genotype at locus *G*_*bi *_on *TG*,



with μ_*WT *_(*s*) = 160 pounds for males or 135 pounds for females and ε_*TG*_(*a*) ~ *N*(0, √0.1), and *S*_*TG*_(*G*_*Tsi*_,*a*) is a sex-specific slope term as follows:

*S*_*TG *_(*G*_*Tsi*_, *a*, *s *= *m*) = (Σ*g*_*si *_+ *r*_*s, TG*_)/20



where *r*_*s*,*TG *_~ *N*(0, √0.1), and *A *= (*a *- 48 + *r*_*f*_), where *r*_*f *_~ *N*(0, √2).

Cholesterol was simulated using a basic linear model, with one sex-limited (female-only) slope gene:

*CHOL*(*a*) = *B*_*CHOL *_(*TG, WT, G*_*bi*_) + (*a *- 20) *S*_*CHOL *_(*s*),

where



with *r*_*CHOL *_and ε_*CHOL*_(*a*) both *N*(0, √0.1), and *S*_*CHOL*_(*s*) is *S*_*CHOL*_(*m*) = (*g*_*s*7 _+ *g*_*s*8_) × (1 + *r*) and *S*_*CHOL*_(*f*) = (*g*_*s*7 _+ *g*_*s*8 _+ *g*_*s*9_) × (1 + *r*), with *r *~ N(0, √0.1). The simulated units on cholesterol were also mg/dl.

#### Blood pressures

Untreated blood pressures were generated first in units of mm Hg by the models:

*SBP *= 115 + *B*_*SBP *_(*G*_*bi*_, *WT, CHOL, SMK, GLUC*) + (*a *- 20) Σ*g*_*si*_

*DBP *= 65 + *B*_*DBP *_(*G*_*bi*_, *SBP*) + ((*a *- 20)/2)Σ*g*_*si*_,

where



and



Both systolic and diastolic blood pressures were used in determining hypertension diagnosis and treatment. However, only SBP was provided with the real data and the simulated replicates, so, consequently, we kept the model for DBP simpler. Hypertension treatment and diagnosis were done separately. Hypertension was diagnosed if SBP > 140 mm Hg or DBP > 90 mm Hg for 2 years in a row, and once the diagnosis was made, it stuck. Hypertension thresholds remained constant, but both the thresholds for treatment and the efficacy of the treatments available changed with time. Before 1960 no treatment was available. From 1960 through 1975, there is a 50% chance (prescription + compliance) of drug treatment if DBP rises above 95. After the initial year of eligibility, there is a 90% chance that an individual remains in the same treatment class. This first treatment lowers SBP by 10 + *N*(0, √2) and DBP by 7 + *N*(0, √1.5). Between 1976 and 1985, this drug treatment remains available and, in addition, we add an "exercise" treatment which those with DBP > 90 have a 50% chance of getting and a 90% chance of remaining in the same treatment class after the initial year of eligibility. The "exercise" treatment lowers SBP by 5 + *N*(0,1) and DBP by 3 + *N*(0, √0.5). In 1986 and after, the first drug treatment is replaced by a treatment with a 75% chance of being administered to those with SBP > 150 or DBP > 95. After the initial year of eligibility, there is a 95% chance of remaining in the treated class, and those untreated have a 50% chance of switching to the treated class. This treatment lowers SBP by 20 + *N*(0, √2) and DBP by 12 + *N*(0, √1.5).

#### Death

Each person had an exponentially increasing probability of dying each year:

δ(*a*) = 10^α*a *^(1 + (*PY *+ *SMK*)/50 + (*e*^(*SBP*-140)/10 ^- 1)/1000 + max (*CHOL *- *R*(*s*),0)/100),

where α = 5 × 10^-7^, *PY *is pack-years, and *R*(*s*) is a sex-specific risk factor of 200 if male and 230 if female. However, we placed the restriction that no person could die before their youngest child was born. If an individual was simulated with an age of death before the birth of their youngest child, we increased the age of death to their age when their youngest child was born.

### Missing data simulation

The missing data pattern in the simulated data set was simulated to resemble the missing data pattern in the real data. In the real data, each visit had a planned subset of the measurements that were to be taken. For each visit in the simulated data, only the planned measurements for that visit are included. A visit is considered to be complete if all the planned measurements were taken.

In the real data, for each visit a subject may have none, some, or all of the planned measurements missing. Only visits with all the planned measurements missing were used to determine the missing data patterns for the simulated data sets. In the simulated data set, a visit is either entirely missing or is complete.

The missing data patterns were summarized into three variables, *H*_*ij *_as predictors of missingness *M*_*ij *_for subject *i *on visit *j*: an indicator of the previous visit being missing; an indicator of the visit two time periods ago being missing; and the proportion of possible visits that are missing. The pattern of missing data for an individual includes all three of these variables. The pattern of missing data for a spouse includes the indicator variables for the last two visits. For parent, and sibling history, only the percentage of missing observations is used.

For each cohort, a logistic model was used to predict the probability of a visit being missing given the subject's missing data pattern, the missing data pattern of first degree relatives, the missing data pattern of the spouse, the measurements at the last nonmissing visit, marital status, being an only child, and visit number. Observations after time of death were not included in the model because these are obviously missing.

For the initial cohort, the previous missing data pattern of the subject, , where  denotes the proportion of missing values up to and including visit *j*, and corresponding values for the subject's spouse (*sp*), and the subject's siblings (*sib*) were predictors. Marital status (*MS*_*ij*_), being an only child (*OC*_*i*_), cholesterol (*CHOL*_*ij*_), weight (*WT*_*ij*_), and visit number (*j*) were also predictors. The following logistic models were created for the original cohort based on the real data (the specific coefficients are listed in Table 4):

1. P(*M*_*ij*_| **M**_*i*,*j*-1_, *CHOL*_*ij*_, *WT*_*ij*_, *OC*_*i*_, *MS*_*ij*_, *j*)

2. P(*M*_*ij*_| **M**_*i*,*j*-1_, **M**_*sp*,*j*_, *HSP*_*ij*_,*CHOL*_*ij*_, *WT*_*ij*_, *OC*_*i*_, *MS*_*ij*_, *j*)

3. P(*M*_*ij*_| **M**_*i*,*j*-1_, **M**_*sib*,*j*_, *CHOL*_*ij*_, *WT*_*ij*_, *OC*_*i*_, *MS*_*ij*_,*j*)

4. P(*M*_*ij*_| **M**_*i*,*j*-1_, **M**_*sp*,*j *__*j*_, **M**_*sib*,*j*_, *CHOL*_*ij*_, *WT*_*ij*_, *OC*_*i*_, *MS*_*ij*_, *j*).

For the offspring cohort, the missing data pattern of the subject, the subject's siblings, and the subject's parents (**M**_*pa *_= (, )), together with indicators **NA**_*pa *_for the availability of parents in the data set, are predictors. Being an only child, cholesterol, weight, and visit number were also predictors. The following models were created for the offspring cohort based on the real data:

5. P(*M*_*ij*_| **M**_*i*,*j*-1_, **M**_*pa*_, **NA**_*i*_, *CHOL*_*ij*_, *WT*_*ij*_, *OC*_*i*_, *j*)

6. P(*M*_*ij*_| **M**_*i*,*j*-1_, **M**_*sib*,*j*_, **NA**_*i*_, **M**_*pa*_, **NA**_*i*_, *CHOL*_*ij*_, *WT*_*ij*_, *OC*_*i*_, *j*).

The models were used to assign missing data patterns as follows: A subject in the original cohort was selected at random from a family and assigned a missing status for Time 3 based on Model 1. (No observations were missing for the first two visits.) The subject's first degree relatives in the original cohort and spouses were then assigned missing values given the already assigned data using Models 2–4. This continues until all subjects in the original cohort were assigned a missing status for Visit 3. The process is repeated for all other visits. Members of the offspring cohort were then assigned missing status. One of the subjects of a sibship was selected at random and assigned a missing value for Time 2 using Model 5. The other siblings were then assigned missing values based on the values of the already assigned siblings (Model 6). This was done for all sibships in the offspring cohort. The process was repeated for all the other times.

Genetic data were collected after a certain time point. Any subject alive at the time when the genetic data were collected had a probability of having genetic data based on how many visits were not missing during the period genetic data was collected. This is similar to what is seen in the real data. In the simulated data set, a subject that had no visits during the time of genetic data collection has no marker data. A subject that has one visit has an 82% chance of having genetic data, and a subject that has more than one visit has an 85% chance of having genetic data.

### Validation

The data were tested at several stages. As the raw trait data were generated, we computed means and variances for each trait and compared the simulated values to those in the real data. We did many graphical comparisons of the simulated data and the real FHS data, particularly with trait vs. age plots. We compared correlation matrices in the simulated data to those in the real data that we were attempting to mimic. We ran linkage detection programs to confirm that we could detect some of the trait genes we simulated. Unfortunately, given the very tight time constraints, we were not able to check every part of the simulation to the degree we would have liked.

Despite our best attempts to check all aspects of the simulation, one serious bug escaped our notice and was detected by Dr. Martin Tobin, affecting the part of the missing data simulation concerning hypertension treatment. This invalidated the data for the purpose of addressing the important question of how to deal with this confounder, but did not affect the genetics of the problem at all. A corrected version of the entire data set was provided to all GAW participants within a month of discovery of the problem. The authors apologize for any inconvenience this may have caused.

## Discussion

In creating this simulated data set, we intended to provide a data set that would facilitate the development of statistical methods for analyzing longitudinal data. This creation involved making a series of compromises — choices about how to model reality and what effects to include given the limited time available. We learned much in simulating this set, and, if we were to start over now and had the same amount of time available, we would make a number of choices slightly differently. Overall, however, we are pleased with the results of our simulation, and we think that having the simulation tightly linked to the real data set greatly increases the value of the simulation.

The simulators and simulation oversight committee agreed that it was of primary importance that the simulation reflect the longitudinal nature of the real data and include some informative missing data patterns. These areas have not been extensively studied in the context of genetic analysis and seem ripe with potential. Many traits have values that change as the body ages, and the contribution of such traits to complex genetic diseases is likely to be important. The missing data patterns included familial missing data patterns and measured covariate effects, neither of which included direct genetic influences. In addition, death also induces an informative missing data pattern with a number of indirect genetic influences on this pattern via SBP and cholesterol. Direct genetic influences and hypertension treatment influences on missing data patterns are something we would have liked to include, but did not simply for lack of time: each additional pattern must be carefully balanced to ensure that enough data is not missing.

The trait model used in this simulation was among the most complex ever used in a GAW simulation, but it still falls short of reality and we had many ideas on improving the model that we were unable to pursue. We considered generating the longitudinal data under a stochastic process, rather than the deterministic model with normally distributed random variables that we used. Unfortunately, we felt that writing a program to do this would take more time than we had. We started out thinking in terms of fairly simple models with genes influencing "baseline" or "slope." There are many other ways to model the effects of trait loci over time when one considers interactions and higher-order effects. Fifty trait loci on eight traits were not enough for all the effects that did occur to us. We would have liked to have many other types of effects, and had genes that influenced smoking, drinking, and play a direct role in treatment effect and death. However, there was some risk that we would lose sight of the longitudinal data focus if we included too many different types of effects.

Both the simulation and the genetic analysis of longitudinal data include many challenges. No simulation can perfectly match the complexity of real data, but we think that this simulation provides enough complexity to enable GAW participants to examine many of these challenges. We hope the results of analyses of this set provide answers to some of these challenges.

## Figures and Tables

**Table 1:** Genome placement, traits affected, and allele frequencies for all model trait loci

Locus Name		Haldane Map Position (cM)				Allele Frequencies			
				
	Chrom.	Male	Female	Sex Ave	Trait(s) affected	A	B	C	D
G_b1_	5	63.07	104.37	80.41	HT	0.40	0.30	0.30	
G_b2_	7	128.43	248.54	184.89	HT	0.70	0.30		
G_b3_	13	95.89	145.21	120.10	HT	0.50	0.50		
G_b4_	13	39.71	41.89	41.75	HT	0.85	0.15		
G_b5_	9	131.04	214.88	169.62	HT	0.70	0.30		
G_b6_	7	27.20	31.23	28.69	HT	0.90	0.10		
G_b7_	5	118.23	207.92	157.98	HT	0.50	0.30	0.20	
G_b8_	21	4.47	33.95	18.25	HT	0.20	0.80		
G_b9_	9	38.69	24.72	30.50	HT	0.50	0.50		
G_b10_	7	92.04	159.96	123.53	HT	0.50	0.30	0.20	
G_b11_	13	57.29	83.34	70.53	WT	0.40	0.30	0.20	0.10
G_b12_	9	18.12	4.66	10.83	HDL, TG, GLUC	0.65	0.35		
G_b13_	9	65.43	105.28	82.76	HDL, TG, GLUC	0.50	0.30	0.10	0.10
G_b14_	1	199.01	398.38	293.26	HDL, TG, GLUC	0.75	0.25		
G_b15_	21	34.93	66.97	49.65	HDL, TG, GLUC	0.45	0.35	0.20	
G_b16_	3	88.06	120.35	102.17	HDL, TG, GLUC	0.50	0.40	0.10	
G_b17_	1	63.06	143.18	100.73	HDL, TG, GLUC	0.40	0.30	0.30	
G_b18_	17	57.79	81.79	67.77	HDL, TG, GLUC	0.60	0.30	0.10	
G_b19_	17	44.99	18.83	31.14	HDL, TG, GLUC	0.70	0.20	0.10	
G_b20_	17	49.99	27.83	38.14	HDL	0.30	0.40	0.30	
G_b21_	11	47.00	42.93	45.14	HDL	0.80	0.20		
G_b22_	5	8.64	2.20	5.33	HDL	0.20	0.40	0.40	
G_b23_	19	107.83	128.20	114.52	TG	0.85	0.15		
G_b24_	15	50.70	37.69	43.84	TG	0.90	0.10		
G_b25_	1	108.85	218.67	160.75	TG	0.20	0.55	0.25	
G_b26_	7	15.77	5.57	10.35	GLUC	0.70	0.20	0.10	
G_b27_	3	48.64	36.61	41.84	GLUC	0.80	0.20		
G_b28_	3	144.79	222.50	180.81	GLUC	0.25	0.25	0.50	
G_b29_	17	55.98	73.57	62.89	GLUC	0.55	0.45		
G_b30_	11	60.03	73.31	66.19	CHOL	0.45	0.35	0.20	
G_b31_	1	114.67	238.40	172.93	CHOL	0.15	0.25	0.60	
G_b32_	15	107.90	146.91	124.76	CHOL	0.50	0.45	0.05	
G_b33_	3	49.10	43.87	45.53	CHOL	0.50	0.40	0.10	
G_b34_	5	126.71	237.14	176.08	SBP	0.70	0.30		
G_b35_	13	65.67	104.81	85.16	SBP	0.50	0.50		
G_b36_	7	44.79	51.35	47.49	SBP	0.80	0.20		
G_b37_	21	10.68	49.64	29.13	DBP	0.85	0.15		
G_b38_	7	6.53	1.02	3.64	DBP	0.90	0.10		
G_s1_	11	43.06	39.41	41.44	WT	0.75	0.25		
G_s2_	7	58.03	68.69	62.69	WT	0.15	0.70	0.15	
G_s3_	5	13.75	3.45	8.46	TG, GLUC	0.40	0.45	0.15	
G_s4_	9	53.45	66.06	58.02	TG, GLUC	0.30	0.30	0.40	
G_s5_	7	97.27	169.70	130.84	TG, GLUC	0.70	0.15	0.15	
G_s6_	21	2.26	17.52	9.04	TG, GLUC	0.60	0.30	0.10	
G_s7_	7	106.64	190.62	145.89	CHOL	0.60	0.30	0.10	
G_s8_	15	63.90	87.03	74.29	CHOL	0.40	0.30	0.30	
G_s9_	21	1.34	3.45	1.92	CHOL (FEMALE)	0.50	0.40	0.10	
G_s10_	21	41.26	68.75	53.59	SBP, DBP	0.70	0.30		
G_s11_	15	9.52	0.00	4.49	SBP, DBP	0.75	0.25		
G_s12_	21	10.95	50.03	29.46	SBP, DBP	0.80	0.20		

**Table 2:** Effects of genotypes at trait loci.

Locus Name	Trait affected			Effect of genotype on trait:									
				
		Variance	Mean	AA	AB	BB	AC	BC	CC	AD	BD	CD	DD
G_b1_	HT	0.4	0	-0.907	-0.475	-0.043	0.173	0.605	1.252				
G_b2_	HT	0.2	0	-0.312	0.088	1.287							
G_b3_	HT	0.1	0	-0.447	0.000	0.447							
G_b4_	HT	0.05	0	-0.071	0.077	1.411							
G_b5_	HT	0.02	0	-0.131	0.087	0.306							
G_b6_	HT	0.02	0	-0.067	0.267	0.600							
G_b7_	HT	0.02	0	-0.072	-0.046	-0.020	-0.059	0.186	0.574				
G_b8_	HT	0.01	0	-0.426	-0.053	0.053							
G_b9_	HT	0.01	0	-0.169	0.074	0.020							
G_b10_	HT	0.01	0	-0.038	-0.028	-0.018	-0.033	0.063	0.466				
G_b11_	WT	0.4	0	-0.564	-0.404	-0.163	1.442	-0.243	-0.002	1.041	0.238	0.640	2.245
G_b12_	HDL	0.2	0	-0.464	0.199	0.862							
	GLUC	0.05	0	0.232	-0.099	-0.431							
	TG	0.1	0	0.328	-0.141	-0.609							
G_b13_	HDL	0.1	0	-0.195	0.125	0.444	-0.163	0.189	2.106	-0.259	-0.003	-0.514	-0.834
	GLUC	0.1	0	0.243	0.018	-0.545	-0.207	-0.657	-0.770	0.356	0.131	-0.095	0.918
	TG	0.1	0	0.191	-0.123	-0.438	-0.186	-0.312	-0.501	0.506	0.065	-0.060	1.766
G_b14_	HDL	0.05	0	-0.141	0.085	0.761							
	GLUC	0.15	0	0.340	-0.416	-0.567							
	TG	0.2	0	0.390	-0.461	-0.745							
G_b15_	HDL	0.02	0	-0.156	-0.006	0.144	-0.081	0.069	0.518				
	GLUC	0.2	0	0.736	-0.095	-0.649	0.182	-0.372	-0.511				
	TG	0.1	0	0.242	-0.161	-0.565	0.444	0.040	-0.363				
G_b16_	HDL	0.02	0	-0.180	0.049	0.201	-0.104	-0.027	0.582				
	GLUC	0.05	0	0.140	0.004	-0.403	0.412	-0.132	-0.268				
	TG	0.02	0	0.213	-0.036	-0.201	-0.118	0.047	0.130				
G_b17_	HDL	0.01	0	0.066	-0.060	-0.186	0.129	0.003	-0.123				
	GLUC	0.05	0	-0.237	-0.183	-0.022	0.032	0.139	0.569				
	TG	0.05	0	-0.339	-0.143	-0.025	0.053	0.211	0.446				
G_b18_	HDL	0.005	0	0.026	-0.012	-0.199	0.063	0.101	-0.086				
	GLUC	0.01	0	-0.082	-0.004	0.114	0.036	0.153	0.699				
	TG	0.01	0	-0.060	0.001	0.032	-0.029	0.245	0.702				
G_b19_	HDL	0.005	0	0.045	-0.092	-0.147	0.073	-0.010	-0.037				
	GLUC	0.01	0	-0.078	0.088	0.155	-0.045	0.254	0.321				
	TG	0.005	0	-0.033	-0.005	0.108	-0.005	0.249	0.390				
G_b20_	HDL	0.15	0	-0.601	-0.204	0.589	-0.403	0.192	0.391				
G_b21_	HDL	0.05	0	-0.166	0.279	0.428							
G_b22_	HDL	0.01	0	-0.142	-0.118	-0.046	-0.094	0.122	0.050				
G_b23_	TG	0.01	0	-0.044	0.077	0.561							
G_b24_	TG	0.005	0	-0.018	0.046	0.659							
G_b25_	TG	0.005	0	-0.221	-0.028	0.049	-0.105	0.010	0.126				
G_b26_	GLUC	0.1	0	-0.212	0.272	0.175	-0.115	0.369	2.208				
G_b27_	GLUC	0.05	0	-0.150	0.207	0.741							
G_b28_	GLUC	0.02	0	-0.237	-0.065	0.108	-0.151	0.022	0.194				
G_b29_	GLUC	0.01	0	-0.128	0.014	0.156							
G_b30_	CHOL	0.2	0	-0.611	0.240	0.362	-0.489	0.483	0.605				
G_b31_	CHOL	0.15	0	-0.825	-0.436	-0.047	-0.630	0.148	0.342				
G_b32_	CHOL	0.1	0	-0.276	-0.140	0.404	0.268	0.676	2.444				
G_b33_	CHOL	0.05	0	-0.117	-0.053	0.012	-0.085	0.528	1.496				
G_b34_	SBP	0.25	0	-0.463	0.309	1.080							
G_b35_	SBP	0.15	0	-0.353	-0.151	0.655							
G_b36_	SBP	0.1	0	-0.236	0.406	0.535							
G_b37_	DBP	0.4	0	-0.376	0.877	2.129							
G_b38_	DBP	0.1	0	-0.118	0.397	2.456							
G_s1_	WT	0.5	0	-0.445	0.267	2.405							
G_s2_	WT	0.4	0	-1.146	-0.652	0.334	-0.159	0.828	2.308				
G_s3_	TG	0.00408	0.058	0	0.05	0.1	0.02	0.2	0.4				
	GLUC	5.36E-05	0.00059	-0.01	-0.002	0.002	0.004	0.01	0.03				
G_s4_	TG	0.00307	0.0633	0	0.01	0.02	0.1	0.12	0.15				
	GLUC	1.58E-05	-0.0003	-0.01	-0.003	-0.002	0	0.002	0.005				
G_s5_	TG	0.00036	0.0446	0.05	0.025	0	0.075	0.04	0.1				
	GLUC	8.77E-07	-7E-05	0	-0.001	-0.004	0.001	-5E-04	0.002				
G_s6_	TG	0.00119	0.09416	0.1	0.12	0.125	0.02	0.05	0				
	GLUC	6.34E-06	0.00045	0	0.002	0.0025	-0.002	-0.001	-0.02				
G_s7_	CHOL	0.36428	0.385	0	0.25	0.5	0.75	2	4				
G_s8_	CHOL	0.07582	0.291	0	0.1	0.3	0.25	0.5	1				
G_s9_	CHOL	0.03208	0.265	0.1	0.2	0.4	0.3	0.7	1				
G_s10_	SBP	0.12424	0.342	0	0.6	1							
	DBP	0.03016	0.138	0	0.2	0.6							
G_s11_	SBP	0.05859	0.1375	0	0.2	1							
	DBP	0.01934	0.10625	0	0.2	0.5							
G_s12_	SBP	0.01574	0.084	0	0.2	0.5							
	DBP	0.06054	0.184	0	0.5	0.6							

**Table 3:** Smoking and drinking probabilities.

Birth Year, Sex	P(SMK| founder)	P(SMK|no parents smoke)	P(SMK| 1+ parents smoke)	P(DRINK| SMK)	P(DRINK| No SMK)	P(DRINK)
before 1930						
male	0.600	0.375	0.750	0.490	0.390	0.450
female	0.200	0.167	0.333	0.180	0.080	0.100
1931–1940						
male	0.600	0.375	0.750	0.540	0.440	0.500
female	0.300	0.231	0.462	0.220	0.120	0.150
1941–1945						
male	0.600	0.375	0.750	0.540	0.440	0.500
female	0.350	0.259	0.519	0.265	0.165	0.200
1946–1950						
male	0.500	0.333	0.667	0.550	0.450	0.500
female	0.350	0.259	0.519	0.265	0.165	0.200
1951–1955						
male	0.450	0.310	0.621	0.655	0.555	0.600
female	0.350	0.259	0.519	0.315	0.215	0.250

**Table 4:** Logistic regression coefficients for the missing data model.

Model	α^A^	*M*_*i*,*j*-1_	*M*_*i*,*j*-2_		*M*_*sp,j*_	*M*_*sp*,*j*-1_	*M*_*sp*,*j*-2_				*NA*_*mo*_	*NA*_*fa*_	*CHOL*_*ij*_	*WT*_*ij*_	*OC*_*i*_	*MS*_*ij*_	Visit (*j*)
1	-3.88	2.390	1.58	1.06									-0.005	0.003	0.093	0.297	0.0256
2	-4.36	2.650	1.85	0.86	3.83	-1.42	-1.13						-0.005	0.003	0.121	0.770	0.0213
3	-3.87	2.380	1.58	1.06				-0.010					-0.005	0.003	0.092	0.297	0.0256
4	-4.35	2.650	1.85	0.86	3.82	-1.14	-1.13	0.054					-0.005	0.003	0.120	0.770	0.0214
5	-1.52	0.919		1.80					0.87	0.88	-0.18	-0.41	-0.005	0.003	0.224		-0.4510
6	-1.71	0.903		1.74	1.710	0.64	0.73	-0.17	-0.31	-0.005	0.003	-0.4490					

^A^α, intercept; *M*_*ij*_, indicator for subject *i*'s visit *j *being missing (with *i *replaced by *sp*, *mo*, *fa*, *sib *for spouse, mother, father, and sib respectively); , average missingness proportion for subject *i *up to and including visit *j *(if the second subscript is omitted, the average is taken over the entire history); *MS*, marital status; *NA*, indicator for parents' being not available in the data set; *OC*, only child; *CHOL*, cholesterol; *WT*, weight.
